# Immune reconstitution in children following chemotherapy for acute leukemia

**DOI:** 10.1002/jha2.27

**Published:** 2020-06-10

**Authors:** Anthony P. Williams, Jessica Bate, Rachael Brooks, Julia Chisholm, Stuart C. Clarke, Elizabeth Dixon, Saul N. Faust, Angeliki Galanopoulou, Paul T. Heath, Thomas Maishman, Susan Mapstone, Soonie R. Patel, Ajay Vora, Sam A. Wilding, Juliet C. Gray

**Affiliations:** ^1^ Faculty of Medicine and Institute for Life Sciences University of Southampton Southampton UK; ^2^ NIHR Southampton Clinical Research Facility NIHR Southampton Biomedical Research Centre and Southampton NIHR CRUK Experimental Cancer Medicine Centre University Hospital Southampton NHS Foundation Trust Southampton UK; ^3^ Department of Paediatric Oncology Royal Marsden Hospital Sutton Surrey; ^4^ Southampton Clinical Trials Unit Southampton UK; ^5^ Paediatric Infectious Diseases Research Group & Vaccine Institute St. George's University of London and St. Georges University Hospitals NHS Trust London UK; ^6^ Paediatric Department Croydon University Hospital Croydon UK; ^7^ Department of Paediatric Haematology Great Ormond Street Hospital London UK

## Abstract

Although survival rates for pediatric acute lymphoblastic leukemia are now excellent, this is at the expense of prolonged chemotherapy regimens. We report the long‐term immune effects in children treated according to the UK Medical Research Council UKALL 2003 protocol. Peripheral blood lymphocyte subsets and immunoglobulin levels were studied in 116 participants, at six time points, during and for 18‐month following treatment, with 30‐39 patients analyzed at each time point.

Total lymphocytes were reduced during maintenance chemotherapy and remained low 18 months following treatment completion. CD4 T cells remained significantly reduced 18 months after treatment, but CD8 cells and natural killer cells recovered to normal values. The fall in naïve B‐cell numbers during maintenance was most marked, but numbers recovered rapidly after cessation of treatment. Memory B cells, particularly nonclass‐switched memory B cells, remained below normal levels 18 months following treatment. All immunoglobulin subclasses were reduced during treatment compared to normal values, with IgM levels most affected.

This study demonstrates that immune reconstitution differs between lymphocyte compartments. Although total B‐cell numbers recover rapidly, disruption of memory/naïve balance persists and T‐cell compartment persist at 18 months. This highlights the impact of modern chemotherapy regimens on immunity, and thus, infectious susceptibility and response to immunization.

## 1 INTRODUCTION

Acute lymphoblastic leukemia (ALL) is the commonest childhood malignancy, with approximately 400 new cases each year in the United Kingdom. Outcome has improved dramatically over the last 30 years, with long‐term survival now in excess of 90% [1, 2, 3]. In the United Kingdom, current regimens entail just over 2 years of chemotherapy for girls, and just over 3 years of treatment for boys. Between 2003 and 2011, the majority of pediatric patients in the United Kingdom with ALL were recruited to the MRC UKALL 2003 trial [4]. This protocol, similar to other treatment regimens internationally, entailed 6‐12 months of relatively intensive blocks of chemotherapy, followed by maintenance chemotherapy (oral 6‐mercaptopurine and methotrexate and four weekly vincristine and steroid pulses) for the remainder of the treatment period. Treatment was stratified according to conventional clinical, cytogenetic, and morphological response criteria, with three treatment regimens (A, B, and C), of increasing intensity.

There have been a number of studies that have reported the immune effects of ALL treatment, but few have comprehensively examined the effects of impact of modern chemotherapy regimens and characterized the immune recovery following cessation of treatment. During the first few months of treatment, children experience significant neutropenia, but this is less common during maintenance chemotherapy [5]. However, lymphopenia, with low levels of B and T cells is common, and is reported to persist for up to 6 months after treatment [6, 7]. B cells have been reported to be more profoundly affected than T cells, with naive B‐cell numbers falling proportionately more than memory B‐cell populations [8, 9, 10]. After treatment, variable rates of reconstitution of B‐cell subpopulations have been reported, with normal counts documented between 3 and 18 months in different studies [5, 9, 10, 11, 12, 13, 14]. Serum levels of immunoglobulin fall during therapy and loss of protective levels of some specific antibodies in previously immunized children are seen [11, 15]. Immunoglobulin levels have been reported to remain low for up to a year after completion of therapy [13]. The reported effects of chemotherapy on T‐cell populations are less consistent, but with more significant effects reported on CD4^+^ T cells and relative modest effects on CD8^+^ T‐cell numbers [7, 8, 9]. Reports on the effects on natural killer (NK) cells are limited and inconsistent [16, 17].

The risk of infection following chemotherapy reflects both loss of pre‐existing immunity (including vaccine immunity) as well as inability to mount new immune responses. Dissecting out the relative importance of these effects is important in determining strategies for reimmunization. It has been reported that children demonstrate adequate responses to reimmunization with booster vaccines 6 months following completion of chemotherapy [18]; and this is current UK practice [19]. However, the timing of reimmunization in these children is largely historical, and it may be that immunization sooner after treatment may be possible, potentially restoring vaccine‐specific immunity earlier.

Here, we describe the immune function of these children, during maintenance chemotherapy and after treatment, to characterize the effects of current ALL treatment regimens. We performed a prospective analysis of peripheral blood lymphocyte subsets and immunoglobulins from children enrolled on a clinical trial, “Investigating the clinical use of 13 valent Pneumococcal‐Conjugate Vaccine in children with ALL” (ISRCTN: 12861513) [20] and treated according to the MRC UKALL 2003 protocol. Analysis was performed at a range of time points from maintenance treatment up to 18‐month following treatment.

## 2 Methods

### 2.1 Study population and study design

The study population consisted of patients recruited to a study assessing the immunogenicity of a 13‐valent pneumococcal conjugate vaccine (PCV‐13) in children with ALL (ISRCTN: 12861513) [20], from which serial blood samples were available for immunological analysis. Patients received leukemia treatment according to the MRC UKALL 2003 trial protocol. The study population comprised of 116 children (1‐18 years of age) receiving first line treatment for ALL, recruited between September 2010 and August 2015. Participant recruitment occurred at eight pediatric oncology centers in the UK (Southampton, Royal Marsden (London), Great Ormond Street (London), Manchester, Cambridge, Bristol, Oxford, and Exeter). Children with concomitant acquired or congenital immunodeficiency, or recent immunosuppressive medication other than UKALL 2003 chemotherapy were excluded. The study was approved by the national ethics committee (REC approval 09/H0504/112) and written informed consent was obtained for all participants.

Chemotherapy treatment details for the UKALL 2003 protocol have been previously published [21].

### 2.2 Lymphocyte subsets and immunoglobulin levels

Blood samples for lymphocyte subset and immunoglobulin analysis were obtained at six different time points: 6 months from last intensive chemotherapy (early maintenance), 18 months from last intensive chemotherapy (late maintenance), 1 month after completion of maintenance chemotherapy (end of treatment), and 6, 12, and 18 months from end of treatment. Samples were obtained from each participant at a maximum of two time points, with numbers at each time point as specified in the figure legends. Lymphocyte subsets and serum immunoglobulins were quantified by local ISO 15189 accredited NHS laboratories in each site, using validated protocols, to assess total numbers of circulating lymphocytes and subsets (Table 1) and serum immunoglobulin and IgG subclass concentrations.

**TABLE 1:** Immunophenotyping markers used to quantify numbers of peripheral blood lymphocyte subsets

Lymphocyte population	Phenotype
T‐cells	CD3+, CD8+
	CD3+, CD4+
Natural Killer Cells	CD56+
B‐cells	CD19+
Naïve B‐cells	CD19+, CD27−, IgM/D+
Memory B‐cell	CD19+, CD27+
Class‐switched Memory B‐cell	CD19+, CD27+, IgM/D−
Nonclass‐switched Memory B‐cell	CD19+, CD27+, IgM/D+

### 2.3 Statistical analysis

Absolute counts for total lymphocytes, total B cells, naïve B cells, unswitched memory B cells, switched memory B cells, CD4 T cells, CD8 T cells, and NK cells were assessed for normality at each time point, using a combination of tests (ie, the Anderson‐Darling, Kolmogorov‐Smirnov, and Cramer‐von Mises tests) along with the graphical representation of the data. Wilcoxon signed‐rank tests for non‐normal data and paired *t*‐tests for normal data were performed, for each time point to assess if the counts differ from those of healthy children (median normal reference value for patients age [22, 23]). For graphical presentation, these counts were expressed as a percentage of median normal reference value for patients’ age [22, 23] (NB. for each patient the following was calculated absolute count divided by median count for normal patients of this age × 100 − a patient with a normal cell count for their age would have a value of 100%), and summarized using Box‐and‐Whisker plots by time.

Total lymphocytes and total B cells values (as a %, as calculated above) were summarized using bar graphs and compared using Kruskal‐Wallis tests (for non‐normal data) and analysis of variance (for normal data) to test for differences among different chemotherapy regimens and between boys and girls.

A two‐sided *P*‐value of <0.05 was deemed statistically significant for all analyses.

Immunoglobulin levels and IgG subclasses were summarized using bar graphs by time and by calculating the percentage of children's results which were within normal ranges for their age [24].

Statistical software SAS 9.4 was used for the analyses.

## 3 Results

### 3.1 Patient demographics

A total of 116 patients had at least one blood sample value available for at least one time point. Consistent with the demographics of ALL, the median age was 6 years (range 2‐17) at the time of recruitment. Demographics of children for each time point are detailed in Table 2. The proportion of patients treated on each Regimen (A, B, C) of UKALL 2003 is reflective of those recruited overall to the UKALL 2003 trial [4].

**TABLE 2:** Patient demographics at each time point

	Early maintenance (Cohort 1 sample 1)	Late maintenance (Cohort 1 sample 2)	End of treatment (Cohort 2 sample 1)	Six months (Cohort 3 sample 1)	12 months (Cohort 2 sample 2)	18 months (Cohort 3 sample 2)
Number of patients with at least one sample available	37	37	39	38	30	32
Gender (Male/Female %)^a^	56.8/43.2	56.8/43.2	56.4/43.6	63.2/36.8	60/40	65.6/34.4
Mean Age (Years, Range)	6.3 (2, 17)	7.4 (3, 18)	8.5 (4, 17)	9.5 (4, 18)	9.7 (5, 18)	10.6 (5,18)
Regimen A (%)	20/37 (54.1)	20/37 (54.1)	24/39 (61.5)	20/38 (52.6)	18/30 (60.0)	17/32 (53.1)
Mean Age (Years, Range)	4.8 (2, 9)	5.9 (3, 10)	6.4 (4, 10)	6.7 (4, 9)	7.6 (5, 11)	7.8 (5,10)
Gender (Male/Female %)^a^	55.0/45.0	55.0/45.0	50.0/50.0	80.0/20.0	55.6/44.4	76.5/23.5
Regimen B (%)	7/37 (18.9)	7/37 (18.9)	10/39 (25.6)	11/38 (28.9)	8/30 (26.7)	9/32 (28.1)
Mean Age (Years, Range)	6.1 (4, 15)	7.3 (5, 16)	13.2 (6, 16)	13.5 (6, 18)	13.9 (7, 17)	13.9 (7,17)
Gender (Male/Female %)^a^	71.4/28.6	71.4/28.6	70.0/30.0	45.5/54.6	75.0/25.0	55.6/44.4
Regimen C (%)	10/37 (27.0)	10/37 (27.0)	5/39 (12.8)	7/38 (18.4)	4/30 (13.3)	6/32 (18.8)
Mean Age (Years, Range)	9.6 (3, 17)	10.6 (4, 18)	9.0 (4, 17)	11.4 (4, 17)	11.3 (6, 18)	13.5 (5,18)
Gender (Male/Female %)^a^	50.0/50.0	50.0/50.0	60.0/40.0	42.9/57.1	50.0/50.0	50.0/50.0

a Percentages rounded to 1 decimal place.

Patient demographics from samples taken at "early" maintenance (approximately 6 months after completion of delayed intensification), "late" maintenance (approximately 18 months after completion of delayed intensification), at the end of treatment (4 weeks from last dose of oral chemotherapy) and 6, 12, and 18 months following completion of treatment.

### 3.2 Lymphocyte compartment

**FIGURE 1:**
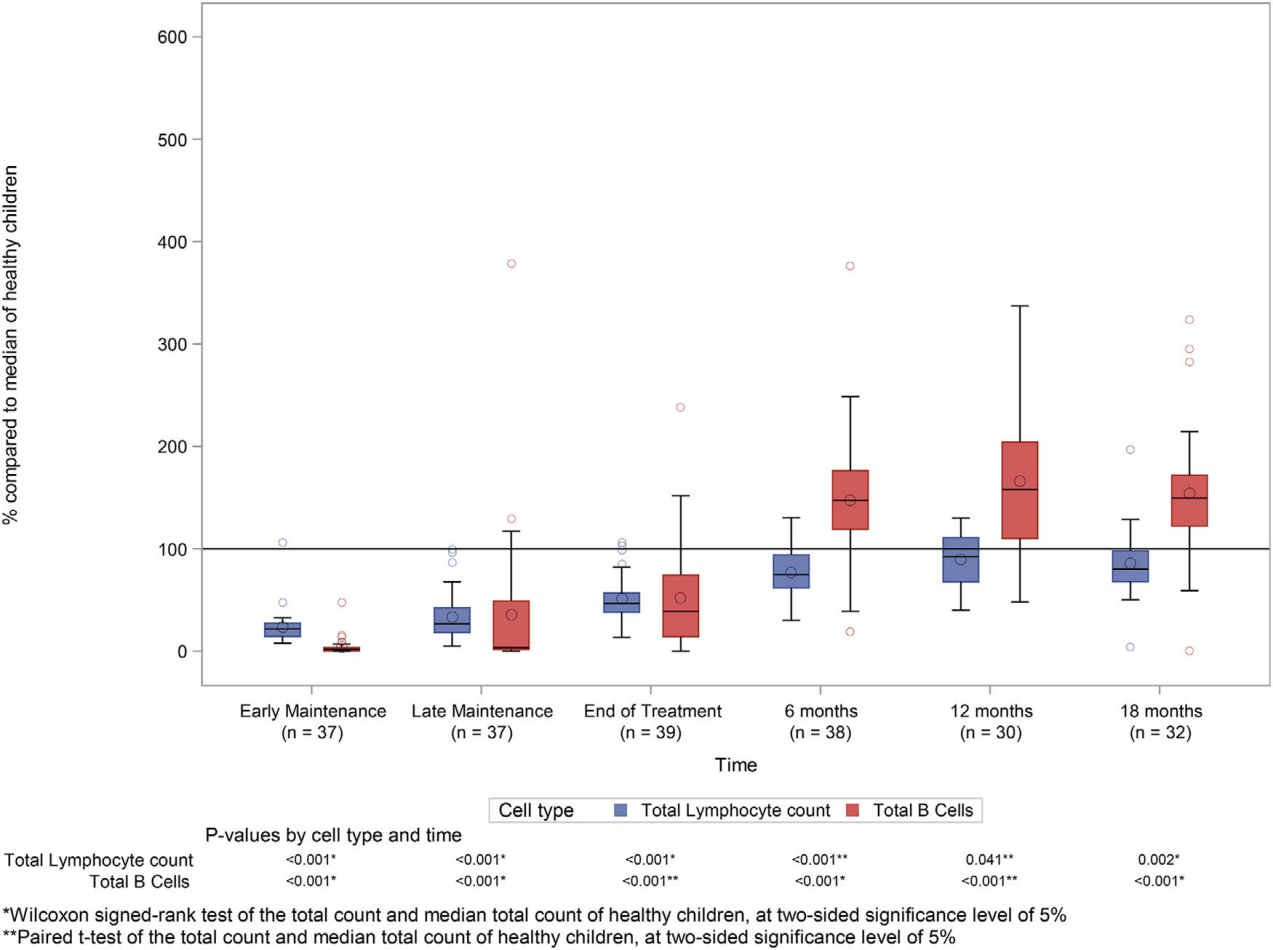
Total lymphocyte and total B‐cell counts during and after treatment. Boxplot showing total lymphocyte (blue) and total B‐cell (CD19+, red) counts at each time point relative to chemotherapy, as percentage of the median of healthy children. Lines within the boxes represent medians and diamonds represent means

In order to assess the effects of chemotherapy on each lymphocytes subset population, absolute numbers were compared to published data for healthy pediatric age groups and expressed as a percentage of median reference range for the relevant age group (as described in detail in statistical analysis). Total lymphocytes counts were significantly reduced during maintenance chemotherapy, with median percentage 21.6% (interquartile range [IQR]: 14.3 to 27.3) and 26.5% (IQR: 18.1 to 42.4) of median reference values for healthy children during early and late maintenance time points, respectively (*P* < 0.001 for both time points) (Figure 1). Although following treatment total lymphocyte numbers gradually improved, counts remained significantly lower than healthy children at 18‐month follow‐up (*P* = 0.002).

### 3.3 Total B cells and naïve B cells

Chemotherapy had a major impact on total B‐cell counts, with a reduction of median percentages of reference values at early and late maintenance to 1.7% (IQR: 0 to 3.4) and 3.4% (IQR: 1.7 to 48.9), respectively (Figure 1). Total B‐cell numbers increased rapidly following cessation of treatment, and although still significantly lower than healthy controls (*P* < 0.001), median percentage counts increased to 38.7% (IQR: 14.1 to 74.2) of reference values by the end of treatment time point, with a rebound to levels that were significantly higher than the normal population by 6 months (median percentage: 147.3%; IQR: 119.0 to 176.2; *P* < 0.001). Levels remain significantly higher than healthy controls 18‐month after treatment (median percentage: 149.5%; IQR: 122.0 to 171.6; *P* < 0.001).

**FIGURE 2:**
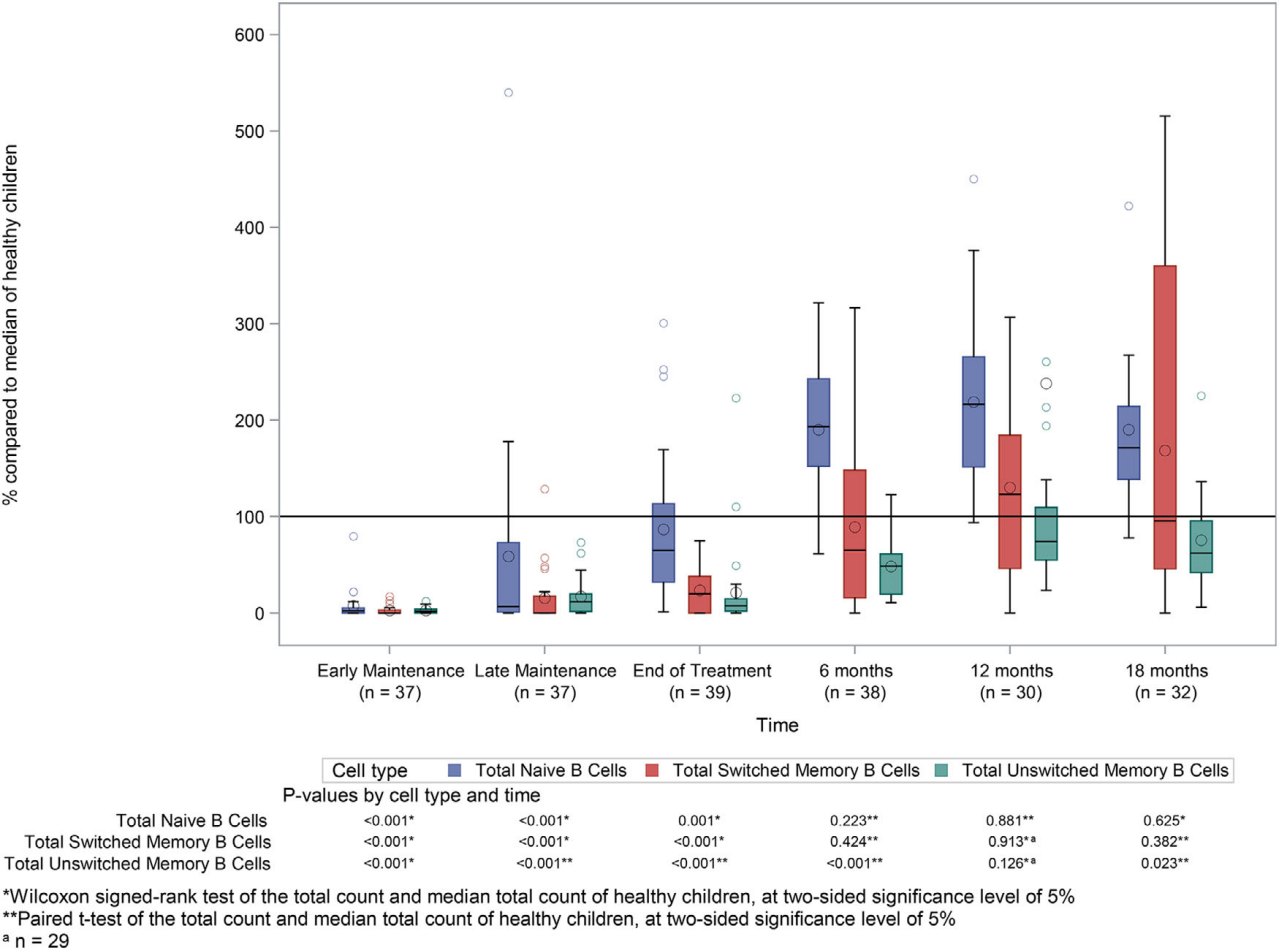
Naïve and memory B‐cell counts during and after treatment. Boxplot showing naïve B cells (blue) and class switched (CD27+IgM/D‐, red) and nonclass switched (CD27+IgM/D+, green) memory B cells. Lines within the boxes represent medians and diamonds represent means (red) and unstitched memory (green). Cell count as a percentage of the median of healthy children, at each time point relative to chemotherapy. Lines within the boxes represent medians and diamonds represent means

Naïve B cells were also severely affected during maintenance chemotherapy, with numbers falling to 2.3% and 6.8% of median reference values at early and late maintenance time points, respectively (Figure 2). Similar to total B‐cell numbers, naïve B cells demonstrated a rapid rebound once treatment was completed, such that they were not significantly different from reference ranges (*P* = 0.088). Again, a rebound recovery was observed, with maximal elevated levels of 216.5% (IQR: 151.4 to 265.5) at 12 months following treatment completion. Numbers remain significantly higher than median values for healthy children even 18 months following cessation of treatment (median percentage: 171.4; IQR: 138.4 to 214.3; *P* < 0.001).

### 3.4 Memory B cells

Memory B‐cell numbers were also significantly affected by chemotherapy with nonclass‐switched memory B‐cell (CD27+, IgM/D+) counts of 1.5% (IQR: 0.0 to 3.9) and 11.7% (IQR: 1.5 to 19.6), and class‐switched memory B cell (CD27+, IgM/D‐) counts of 0.0% (IQR: 0.0 to 3.0) and 0.1% (IQR: 0.0 to 17.2) of the medians of healthy controls at early and late maintenance, respectively (Figure 2). Memory B‐cell recovery was slower than that of naïve B cells, and memory B cells did not demonstrate a rebound increase to higher than normal levels following treatment. Class‐switched memory B‐cell counts recovered steadily following treatment such that they were not significantly different from healthy controls by 6 months from the end of treatment (*P* = 0.424). Nonclass‐switched memory B‐cells counts (CD27+, IgM/D+) recovered slowly following treatment and remained persistently subnormal even at 18‐month follow‐up (*P* = 0.023).

### 3.5 Immunoglobulins

Functionality of B cells, as indicated by circulating levels of immunoglobulins was also affected by chemotherapy (Supporting information Figure 1 and Table 1). Total IgG levels were outside the age‐specific reference ranges in 52.6% and 43.2% of patients during early and late maintenance respectively, and were still low for 3.5% of children 18 months after completing treatment. Of the IgG subclasses, IgG1 and IgG2 were affected most (Supporting Information Figure 2 and Table 1). IgM levels were also significantly affected, and 13.8% of patients had levels persistently low levels 18 months after completion of treatment.

### 3.6 T‐cells CD4^+^ and CD8^+^

**FIGURE 3:**
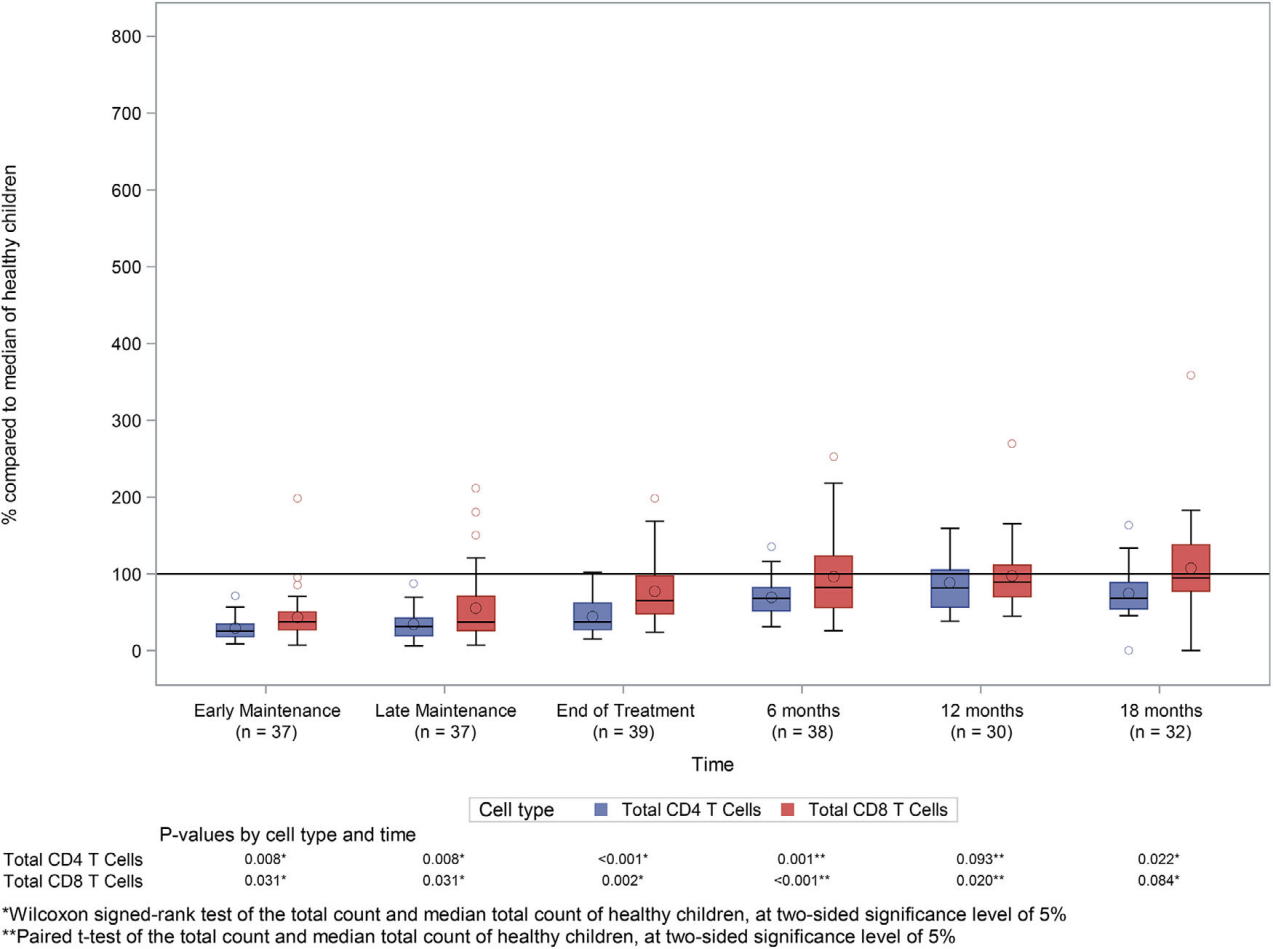
CD4 and CD8 T‐cell counts during and after treatment. Boxplots showing: (A) CD4+ve and (B) CD8+ve T‐cell counts as a percentage of the median of healthy children by time relative to chemotherapy. Lines within the boxes represent medians and diamonds represent means

Both CD4^+^ and CD8^+^ T‐cell subsets were affected by chemotherapy (Figure 3). CD4^+^ T‐cell numbers of 25.2% (IQR: 18.1 to 34.5) and 31.3% (IQR: 19.2 to 42.2) of median reference values were observed in patients during early and late maintenance therapy respectively, and levels were still significantly low 18 months following completion of treatment (*P* < 0.001). CD8^+^ T‐cell counts were less profoundly affected, with counts of 37.6% (IQR: 27.1 to 50.1) and 37.1% (IQR: 26.1 to 70.6) of median reference values seen in early and late maintenance time points respectively, and recovery to normal values by 6 months after treatment.

### 3.7 Natural killer cells

Similar to helper and cytotoxic T cells, NK cell counts were significantly reduced during maintenance chemotherapy to below 30% of normal values at both time points (Supporting information Figure 3). At end of treatment and during follow‐up, cell counts steadily improved but were still significantly low 6 months following treatment cessation (*P* = 0.014). Circulating NK cell numbers were not significantly abnormal by 12 and 18 months after treatment (*P* = 0.526 and 0.254, respectively).

### 3.8 Effect of age on immune recovery

There did not appear to be any substantial differences in immune recovery between different age groups, although children aged over 10 appeared to show higher levels of immune recovery at the 18‐month time point, in comparison with younger children (see Supporting Information Figures 4‐8).

### 3.9 Comparison of different chemotherapy regimens

**FIGURE 4:**
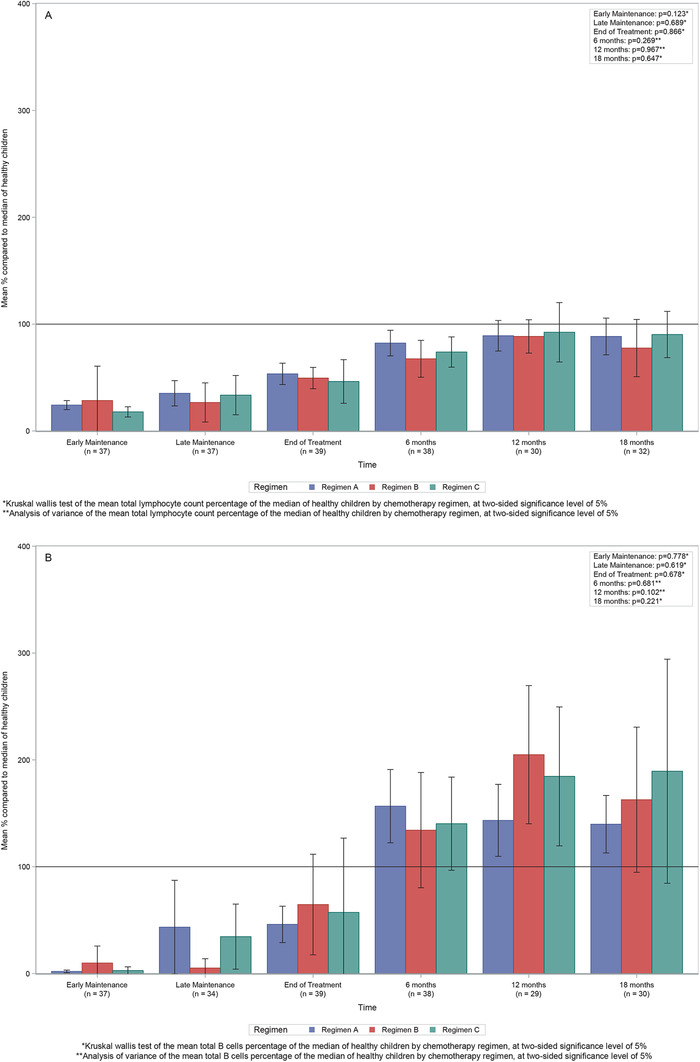
Comparison of different chemotherapy regimens. Bar graphs showing (A) Mean total lymphocyte count percentage of the median of healthy children by chemotherapy regimen. (B) Mean total B‐cells (CD19+) percentage of the median of healthy children by chemotherapy regimen. Lines represent the confidence limits of the means

The UKALL 2003 protocol includes treatment stratification based on established risk factors, such that patients receive one of three different regimens, A, B or C, with increasing intensity. Comparisons were made between the three chemotherapy regimes in the UKALL 2003 protocol for the total lymphocytes and B cells and no significant differences were observed (Figure 4).

### 3.10 Comparison of boys and girls

**FIGURE 5:**
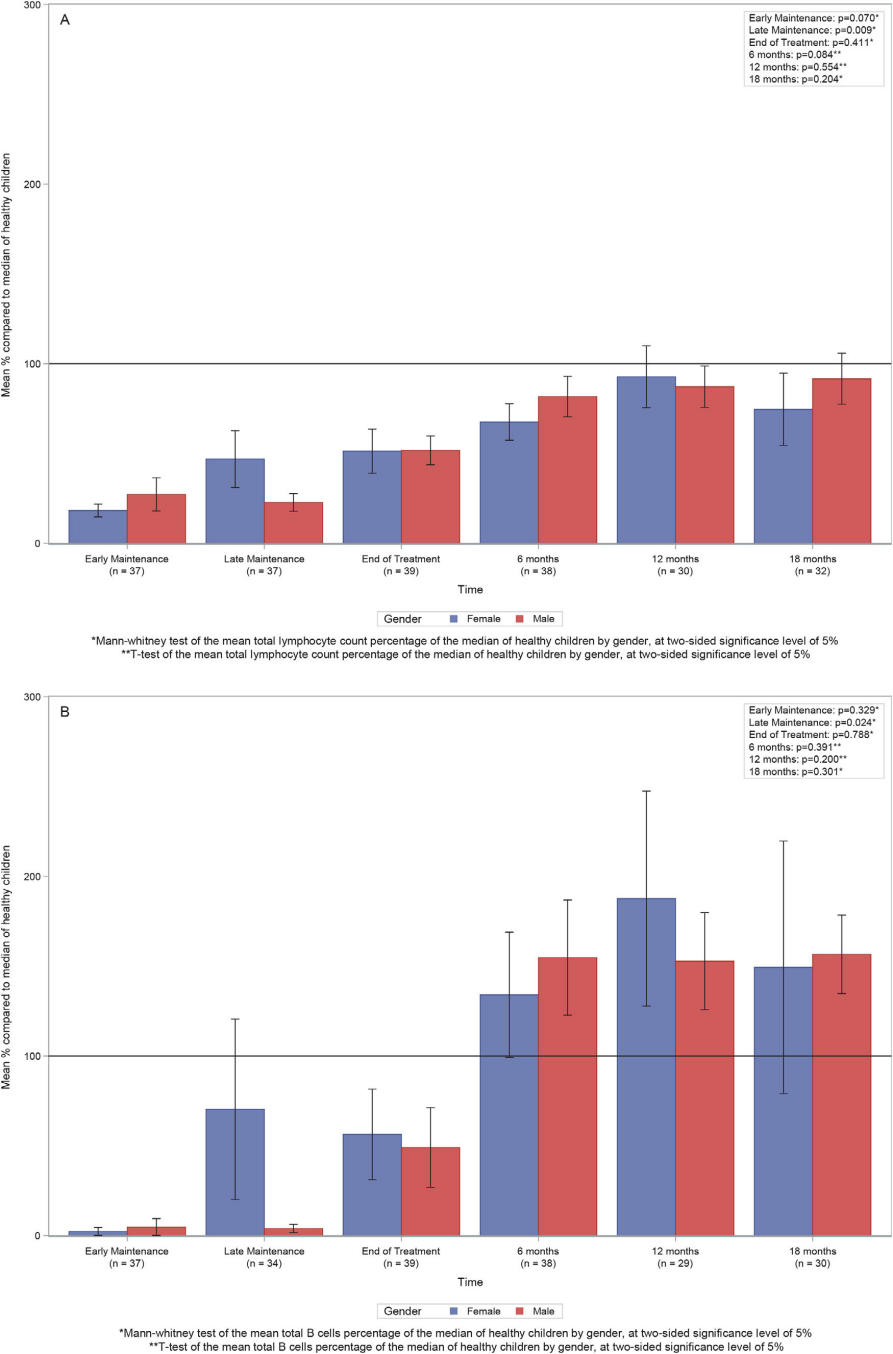
Comparison of immune effects of treatment in boys and girls. Bar graphs showing (A) Mean total lymphocyte count percentage of the median of healthy children by chemotherapy regimen. (B) Mean total B‐cells (CD19+) percentage of the median of healthy children by gender. Lines represent the confidence limits of the means

In view of the fact that boys receive significantly longer treatment than girls (3 years compared to 2 years maintenance therapy), comparisons were made between immune recovery in boys and girls. Significant differences in the immune recovery between boys and girls were observed only at late maintenance for both lymphocytes and B cells (*P* = 0.009 and 0.024, respectively) (Figure 5), but levels following completion of treatment were similar.

## 4 Discussion

In this prospective study, we demonstrated the impact of a contemporary chemotherapy regimen for ALL on long‐term immune reconstitution in a large pediatric patient cohort.

During maintenance chemotherapy, B‐cell counts were severely suppressed, but exhibited relatively rapid recovery following treatment completion. Although naïve and memory B‐cells were similarly affected by chemotherapy in all risk groups, indicative of severe immunosuppression, recovery of the naïve population after chemotherapy was much more rapid, six months after treatment cessation, rebound recovery of total B‐cells counts was noted. Numbers peaked at 1 year after end of treatment. Recovery was attributable to a rebound proliferation of naïve B cells, demonstrative of bone marrow recovery and subsequent high‐bone marrow output. During reconstitution, naïve B cells accounted for a greater proportion of total B‐cell counts than memory B cells. In contrast to naive cells, memory B‐cell counts remained subnormal even at 18 months following treatment cessation. This change in B‐cell compartment distribution is in line with previous reports and the proportions of B cells are similar to those found in infants [4, 5, 16].

Memory B‐cells subsets, particularly nonclass‐switched memory B cells, were found to be profoundly affected, and were 0% of age‐matched median values throughout maintenance therapy. Immunoglobulins were affected by chemotherapy; in particular IgM levels were below the normal range for all children during early maintenance and remained persistently low in a quarter of children 12 months after completion of treatment. Importantly, despite rebound recovery of B cells following treatment cessation, key memory B‐cell subsets and immunoglobulins remained subnormal even after 18 months.

Relative to the B‐cell compartment, the T‐cell compartment was less affected by active chemotherapy but demonstrated a slower reconstitution following treatment cessation. Congruent with earlier reports, CD4^+^ T cells were most severely affected by chemotherapy compared to CD8^+^ T cells [5]. CD8^+^ T‐cell subsets steadily increased following treatment completion but remained subnormal at end of follow‐up; suppression of CD4^+^ T cells persisted even at 18 months. Similar to helper T cells, NK cells were reduced during chemotherapy and numbers remained incomplete at 18 months after chemotherapy treatment.

All pediatric ALL treatment protocols include a prolonged phase of maintenance chemotherapy. In some protocols this consists solely of oral chemotherapy agents (eg, 6‐mercaptopurine and methotrexate) and in others it also includes regular pulsed oral corticosteroids and vincristine. During this time, children are generally well and reintegrating into social and school life. Despite the fact that doses of chemotherapy are titrated to avoid significant neutropenia, 20% of all infection‐related deaths occurred during this phase of treatment [25]. Patients treated according to UKALL 2003, all received a dose of vincristine and a 5‐day pulse of dexamethasone every 4 weeks, in addition to daily oral 6‐mercaptopurine and weekly oral methotrexate, for the duration of maintenance therapy. It is unclear how much each of these agents contribute to immunosuppression, but it has been suggested that the inclusion of dexamethasone may significantly increase infection rates during this phase of treatment [26]. A 2010 systematic review suggested that vincristine and dexamethasone pulses may not be essential to achieve the current excellent event‐free survival [27] and the UKALL 2011 clinical trial (ISRCTN64515327) is specifically addressing this question. The removal of dexamethasone from maintenance therapy may lessen the degree of immunosuppression, improving immune function and reducing infectious morbidity and mortality.

Although there is a considerable range of treatment intensity (Regimen A, B, and C), we did not see any significant impact of this on the degree or duration of immunosuppression. However, much of the intensity variation may affect myelosuppression, and duration of neutropenia, rather than immunosuppression and lymphopenia. Therefore, a reduction in the exposure to more immunosuppressive agents (eg, by removing corticosteroids from maintenance treatment) may have an impact on immunosuppression and infection rates. Indeed, the Dutch Oncology Group has reported that reduction in intensity of maintenance treatment was associated with reduced infectious complications [28]. The data presented here will provide a comparator for future studies to assess the impact of such reductions. Similarly, we did not see any significant difference between the degree of immunosuppression in boys and girls, despite the significantly longer treatment duration in boys. This suggests that the duration of maintenance therapy may be less important than the actual drugs received, in terms of the degree of suppression and time for recovery.

Supportive care for reducing infection‐related mortality is critical [25]. All patients on the UKALL 2003 trial for example, received pneumocystis prophylaxis and no mortalities from this infection were identified. The risk of some other specific infections is also reported to be high, with one study describing the relative risk of invasive pneumococcal infection to be 50‐fold that of healthy children [29]. No prophylactic measures are taken to reduce such infections. Options for this would include regular antimicrobial prophylaxis or immunization. Immunization during treatment is an attractive option, given the duration of risk, but may not be feasible given the degree of B‐cell suppression observed in this study, even with highly immunogenic conjugate vaccines. However, the rapid recovery in naïve B cells observed following cessation of treatment may suggest that very early (eg, 1 month following completion of treatment) immunization may be possible, rather than the conventional recommendation of 6 months later. This is supported by the results of our study of immunization with PCV‐13 in children with ALL, from which these current samples were obtained, that demonstrated protective levels of immunity could be achieved by immunization 1 month following maintenance treatment [20].

In summary, we have clearly demonstrated that the immune system is significantly impaired during treatment during UKALL 2003 therapy and that full recovery may takes up to at least 18 months following treatment. Although the B‐cell compartment is particularly severely affected by chemotherapy, there is a rapid rebound proliferative increase in naïve B cells after treatment. This, together with data we have recently published demonstrating protective responses to PCV‐13 immunization given 1 month after cessation of treatment in this population, suggests that early reimmunization may be feasible [20].

## Author's contribution

JG was Chief Investigator for the clinical trial. Study concept, trial design, and funding application were conducted by AW, JB, JC, SC, SF, PH, and JG. Trial conduct and management was overseen by ED and JG. Patient recruitment, blood sampling, and data collection were conducted by SM. RB, TM, and AG conducted statistical analysis. The manuscript was primarily written by AW, RB, and JG; all authors reviewed final manuscript.

## Supporting information

SUPPORTING INFORMATION

SUPPORTING INFORMATION

SUPPORTING INFORMATION

SUPPORTING INFORMATION

SUPPORTING INFORMATION

SUPPORTING INFORMATION

SUPPORTING INFORMATION

SUPPORTING INFORMATION

SUPPORTING INFORMATION

## Data Availability

The data that support the findings of this study are available from the corresponding author upon reasonable request.
