# Supplementary figures and images for: Immune reconstitution in children following chemotherapy for acute leukemia
Source: EJHaem. 2020 Jun 10;1(1):142–51. doi: 10.1002/jha2.27 (PMC9176016; doi:10.1002/jha2.27)

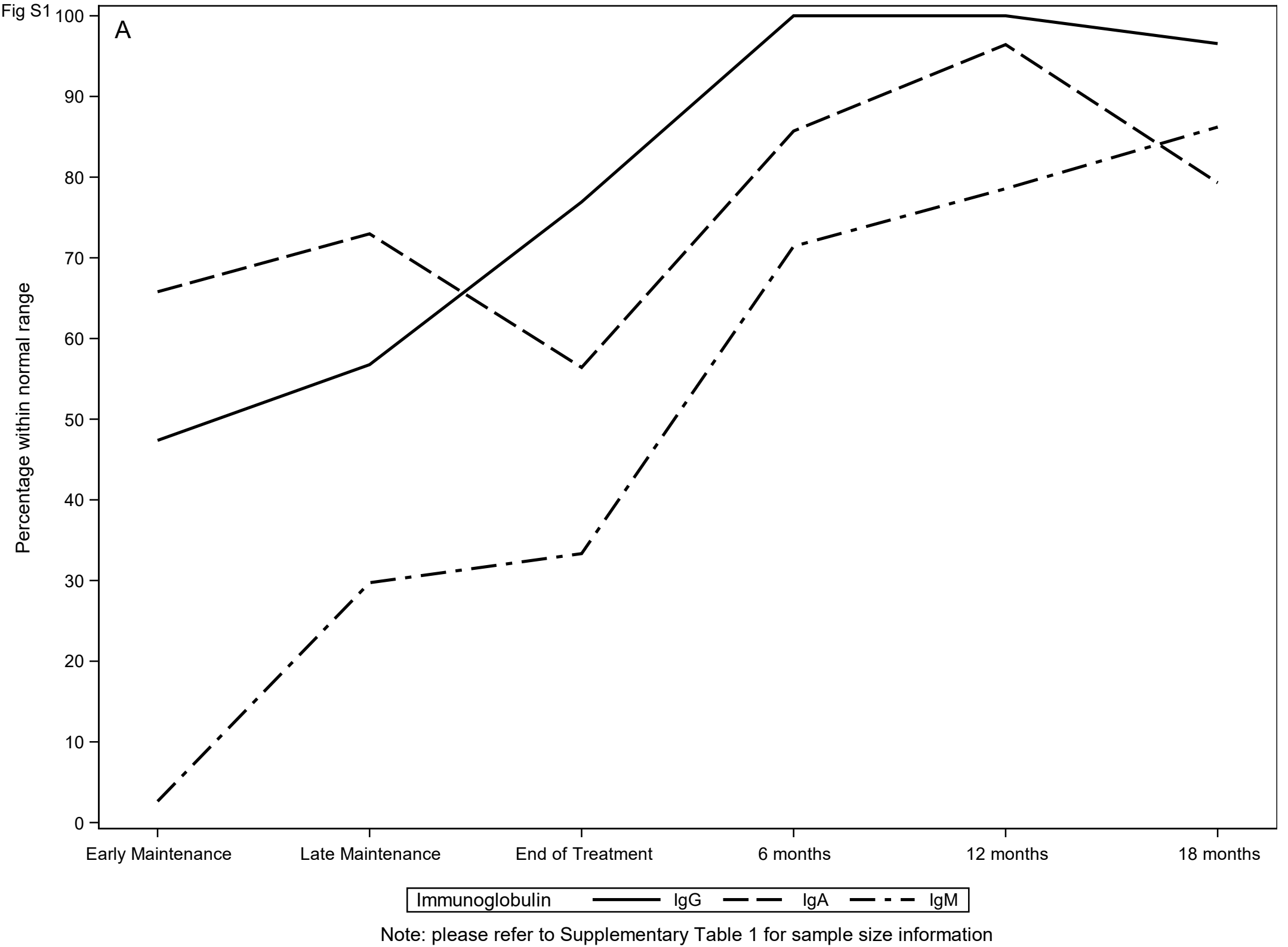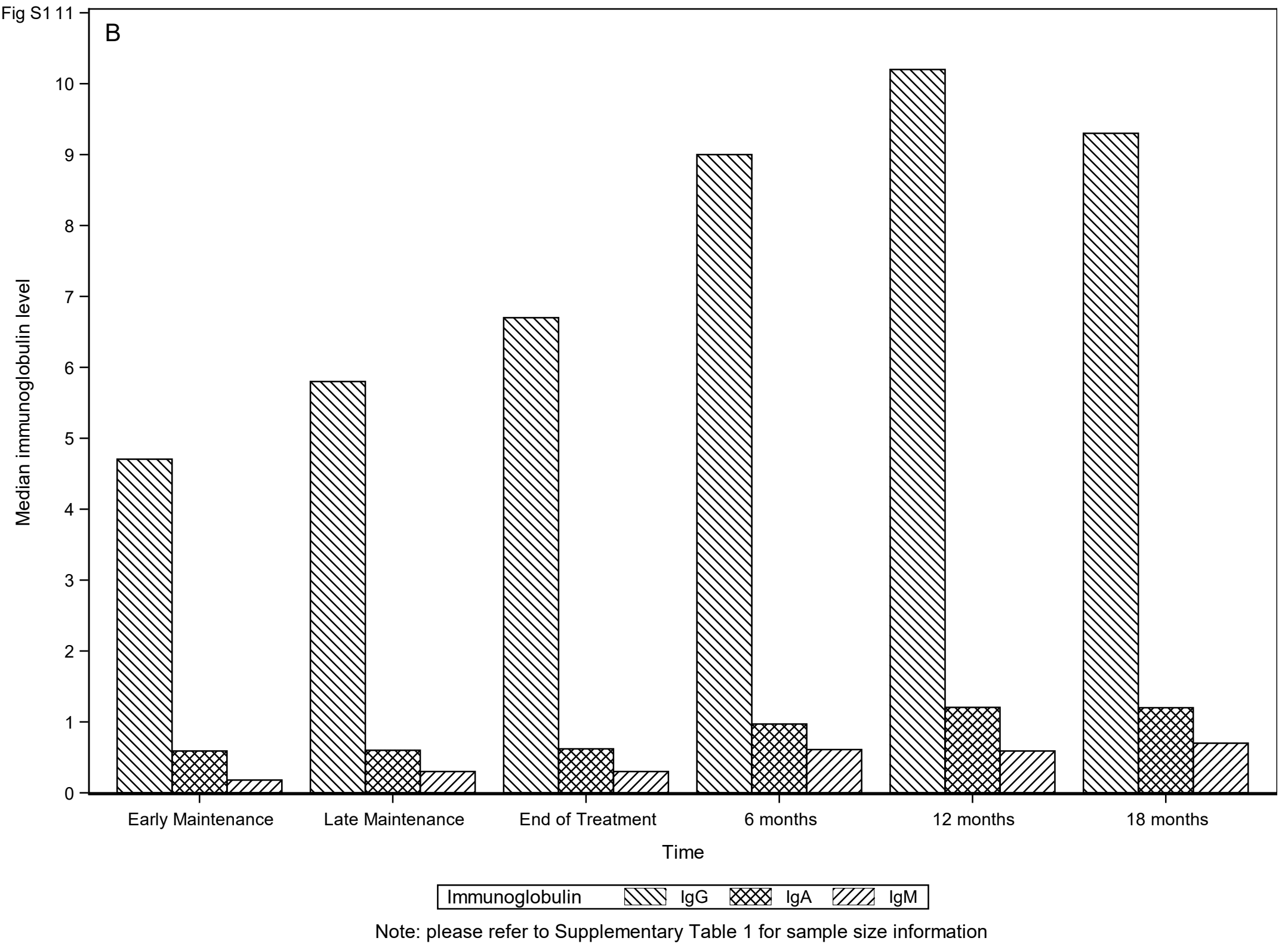

Supplement: Supplementary file 1 — SUPPORTING INFORMATION [file JHA2-1-142-s005.pdf]

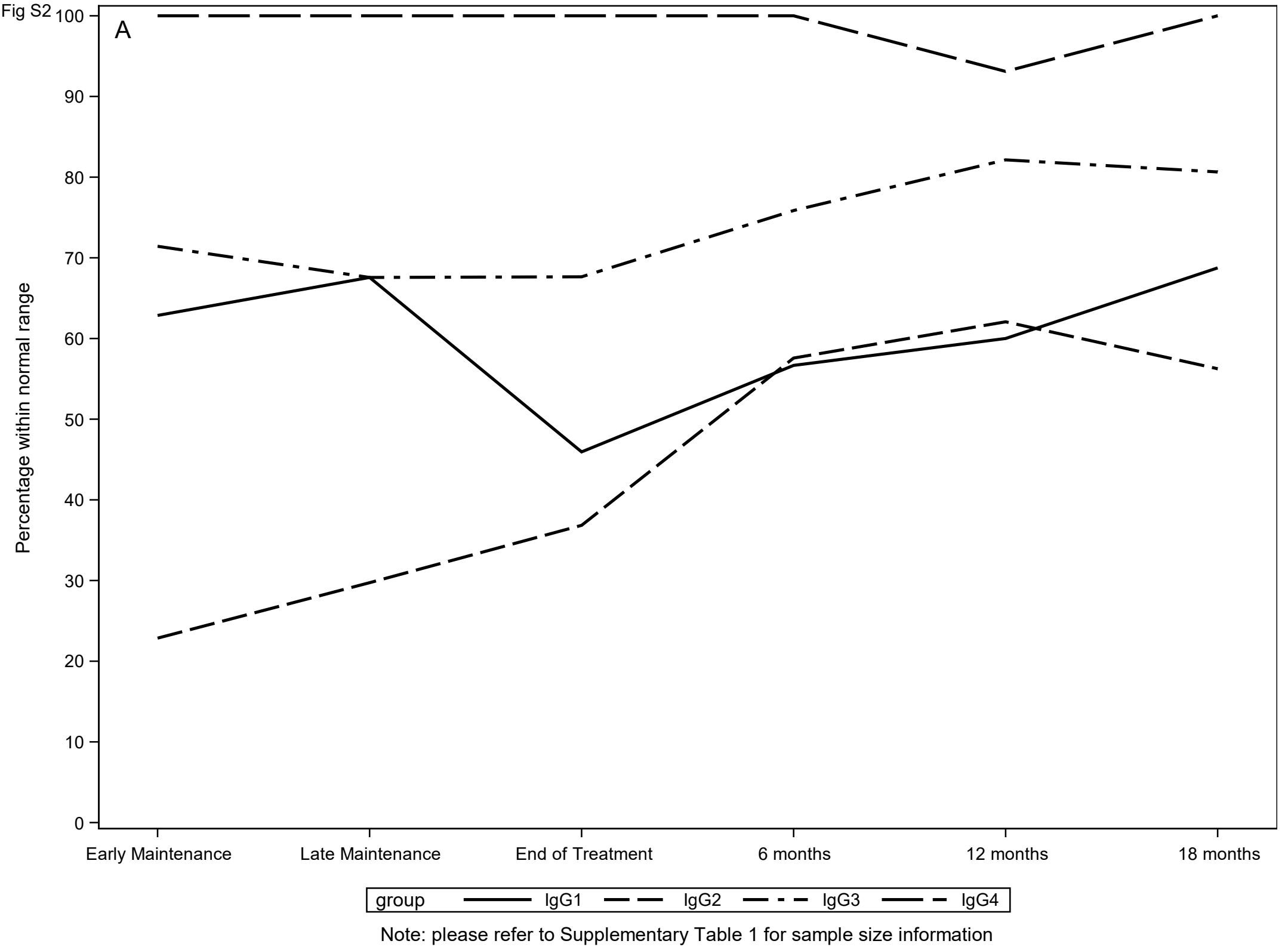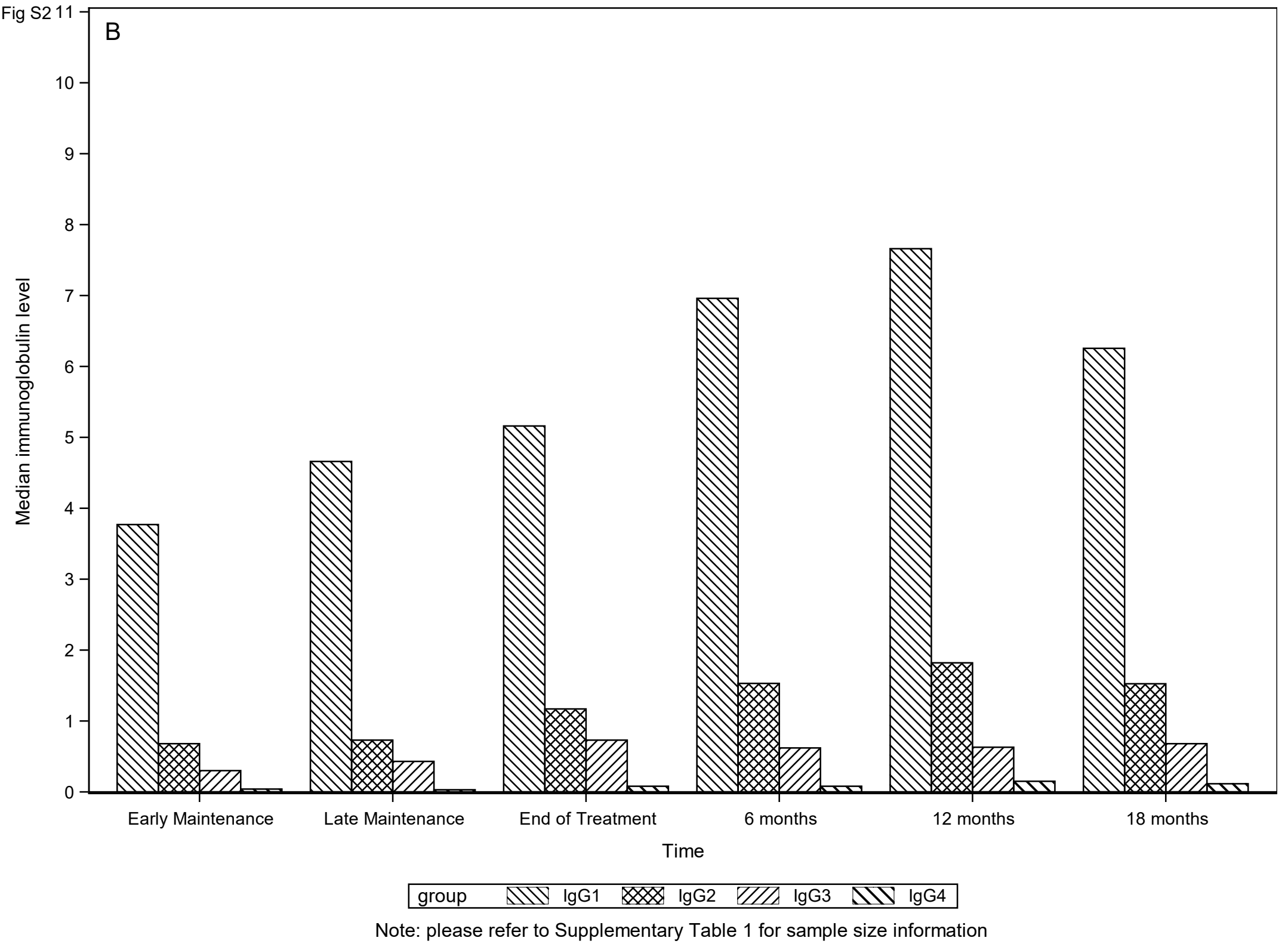

Supplement: Supplementary file 2 — SUPPORTING INFORMATION [file JHA2-1-142-s008.pdf]

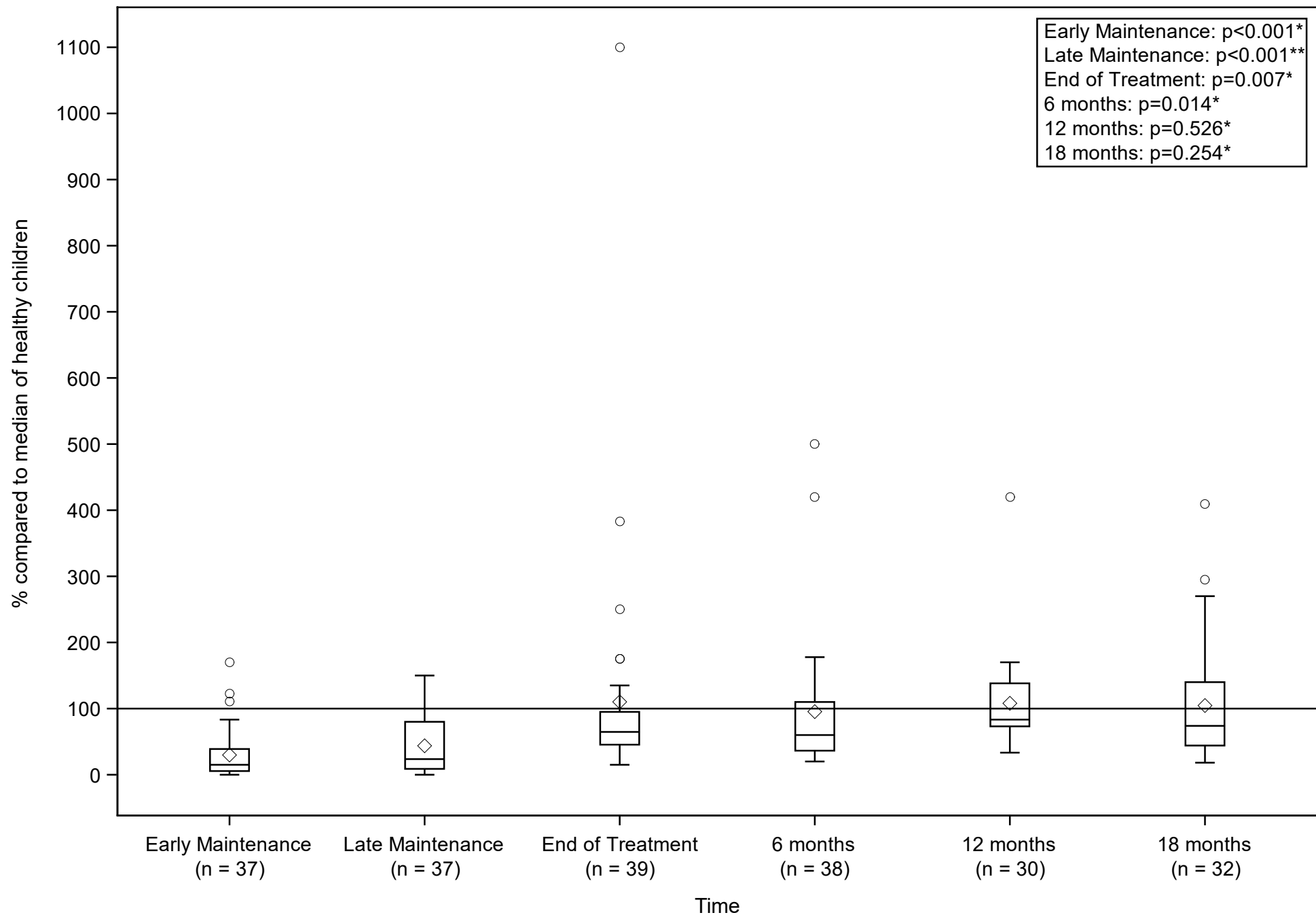

Supplement: Supplementary file 3 — SUPPORTING INFORMATION [file JHA2-1-142-s001.pdf]

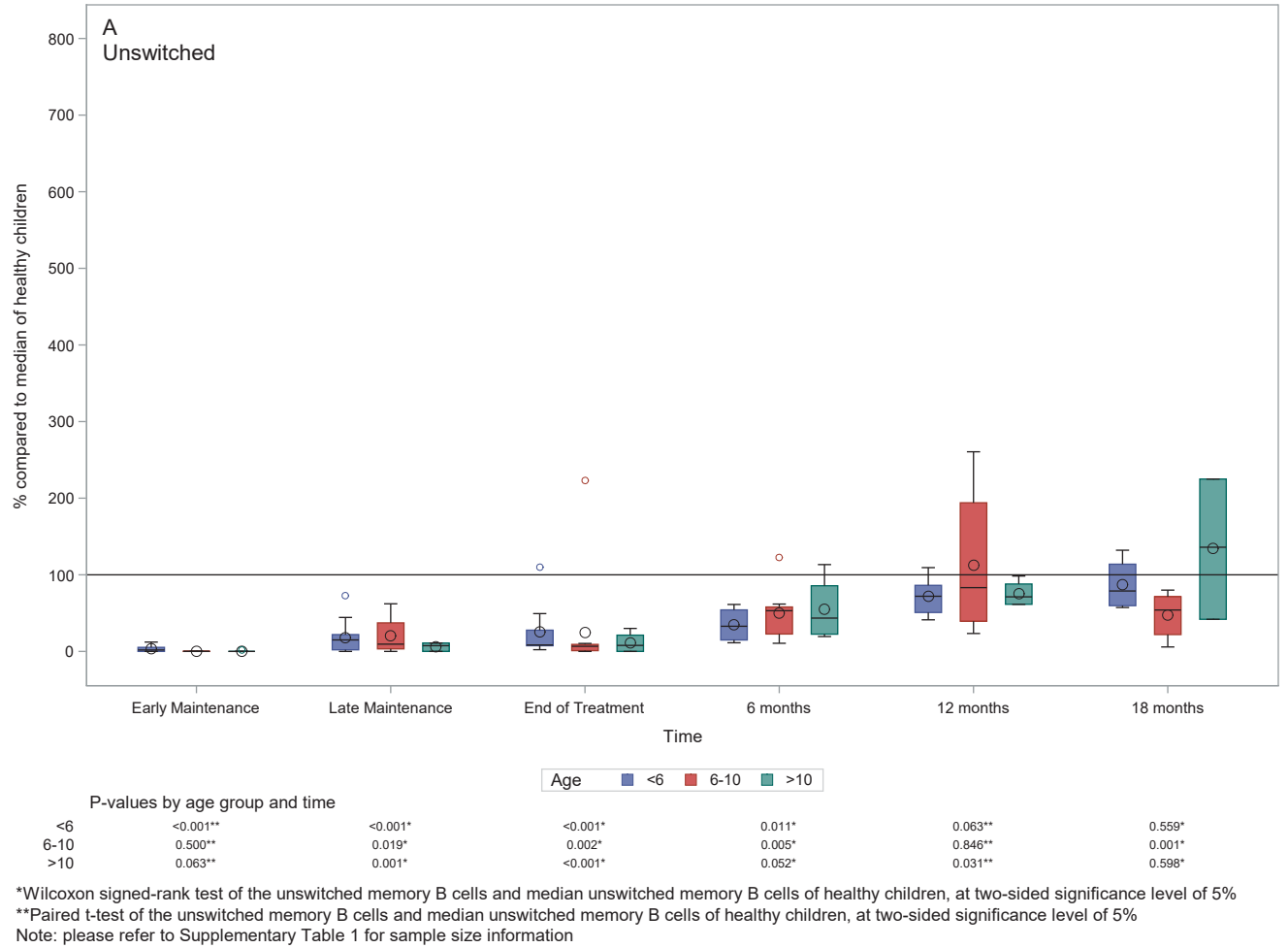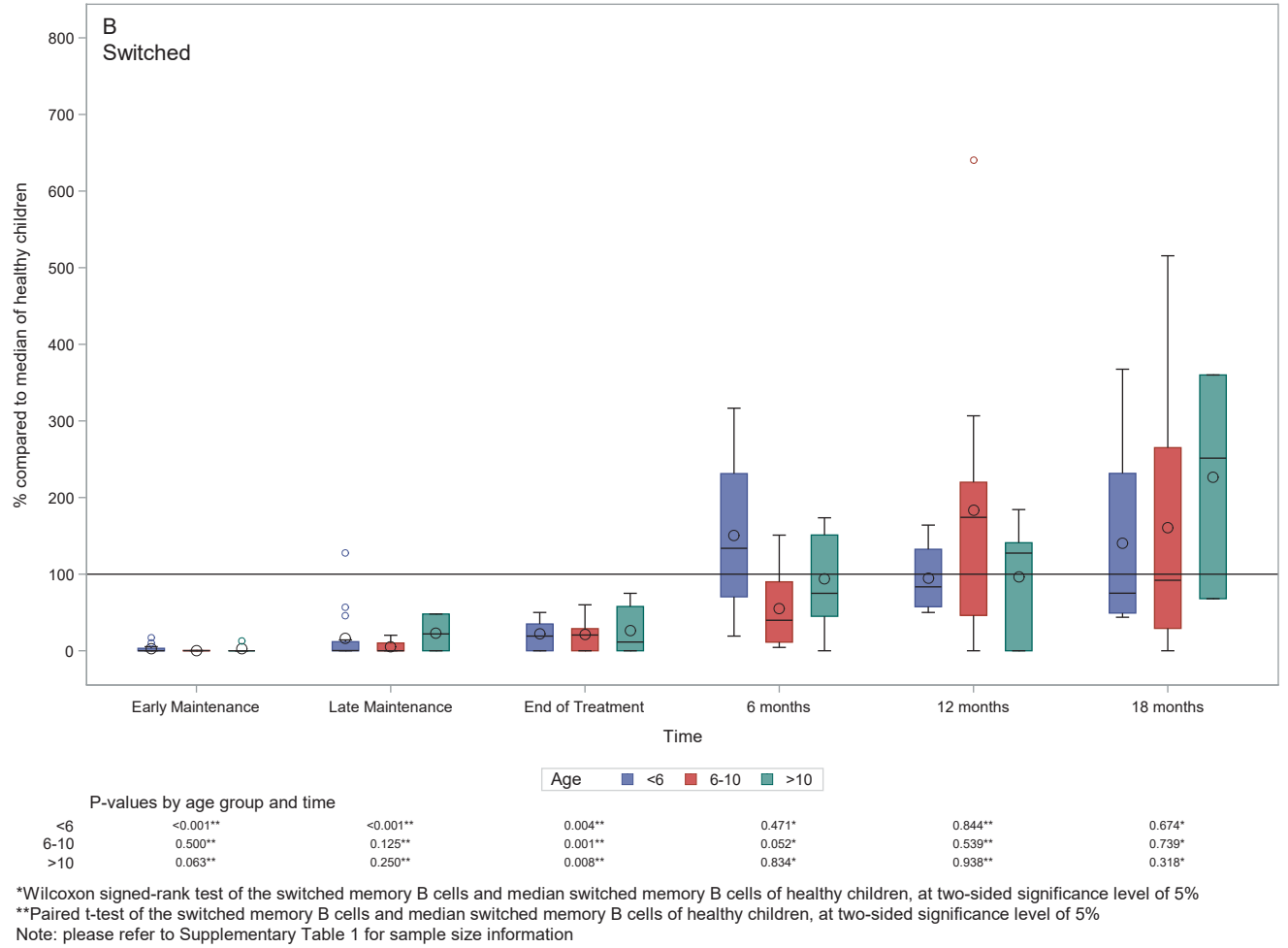

Supplement: Supplementary file 7 — SUPPORTING INFORMATION [file JHA2-1-142-s009.pdf]
