# Supplementary material for: Immune reconstitution in children following chemotherapy for acute leukemia
Source: EJHaem. 2020 Jun 10;1(1):142–51. doi: 10.1002/jha2.27 (PMC9176016; doi:10.1002/jha2.27)
Supplement: Supplementary file 4 — SUPPORTING INFORMATION [file JHA2-1-142-s002.pdf]

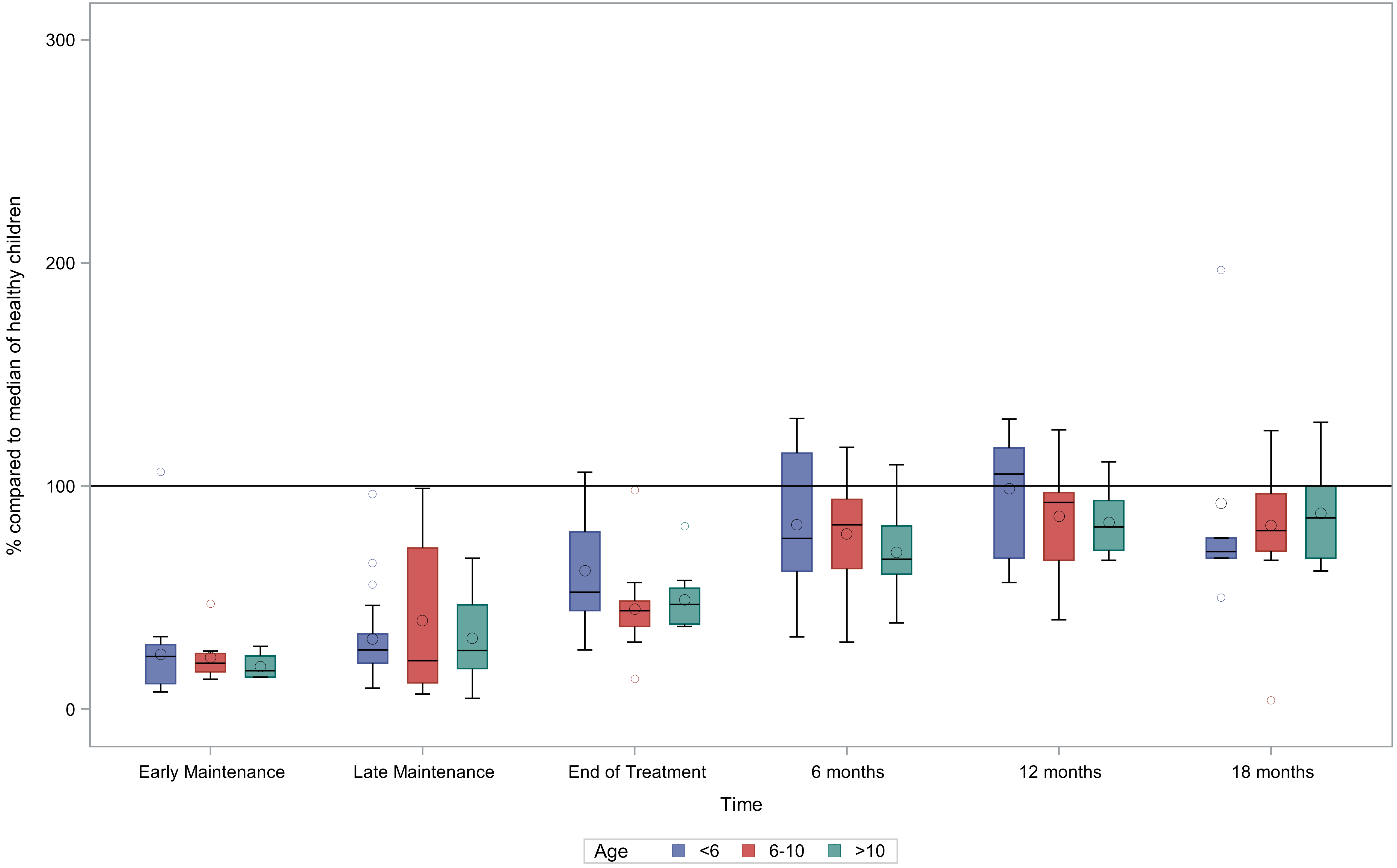

P-values by age group and time

	Early Maintenance	Late Maintenance	End of Treatment	6 months	12 months	18 months
<6	<0.001*	<0.001*	0.001*	0.223**	0.881**	0.625*
6-10	0.008*	0.008*	<0.001*	0.001**	0.093**	0.022*
>10	0.031*	0.031*	0.002*	<0.001**	0.020**	0.084*

*Wilcoxon signed-rank test of the total lymphocyte count and median total lymphocyte count of healthy children, at two-sided significance level of 5%

**Paired t-test of the total lymphocyte count and median total lymphocyte count of healthy children, at two-sided significance level of 5%

Note: please refer to Supplementary Table 1 for sample size information
