# Supplementary material for: Immune reconstitution in children following chemotherapy for acute leukemia
Source: EJHaem. 2020 Jun 10;1(1):142–51. doi: 10.1002/jha2.27 (PMC9176016; doi:10.1002/jha2.27)
Supplement: Supplementary file 5 — SUPPORTING INFORMATION [file JHA2-1-142-s003.pdf]

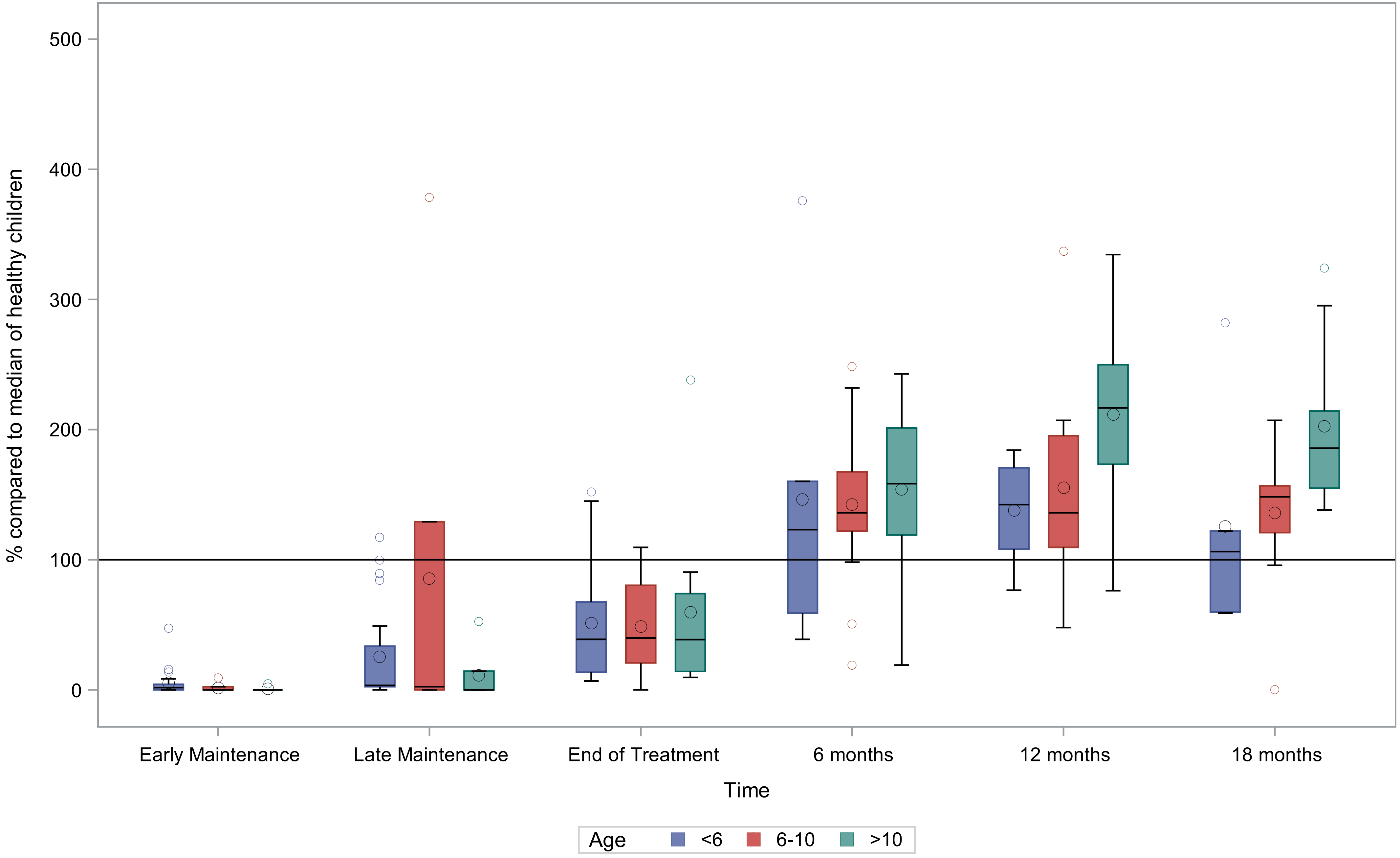

P-values by age group and time

	Early Maintenance	Late Maintenance	End of Treatment	6 months	12 months	18 months
<6	<0.001*	<0.001*	0.003**	0.469*	0.026**	0.999*
6-10	0.008*	0.453*	<0.001**	0.009*	0.017**	0.005*
>10	0.031*	0.031*	0.052**	0.016*	0.009**	0.004*

*Wilcoxon signed-rank test of the total B cells and median total B cells of healthy children, at two-sided significance level of 5%

**Paired t-test of the total B cells and median total B cells of healthy children, at two-sided significance level of 5%

Note: please refer to Supplementary Table 1 for sample size information
