# Supplementary material for: Immune reconstitution in children following chemotherapy for acute leukemia
Source: EJHaem. 2020 Jun 10;1(1):142–51. doi: 10.1002/jha2.27 (PMC9176016; doi:10.1002/jha2.27)
Supplement: Supplementary file 6 — SUPPORTING INFORMATION [file JHA2-1-142-s004.pdf]

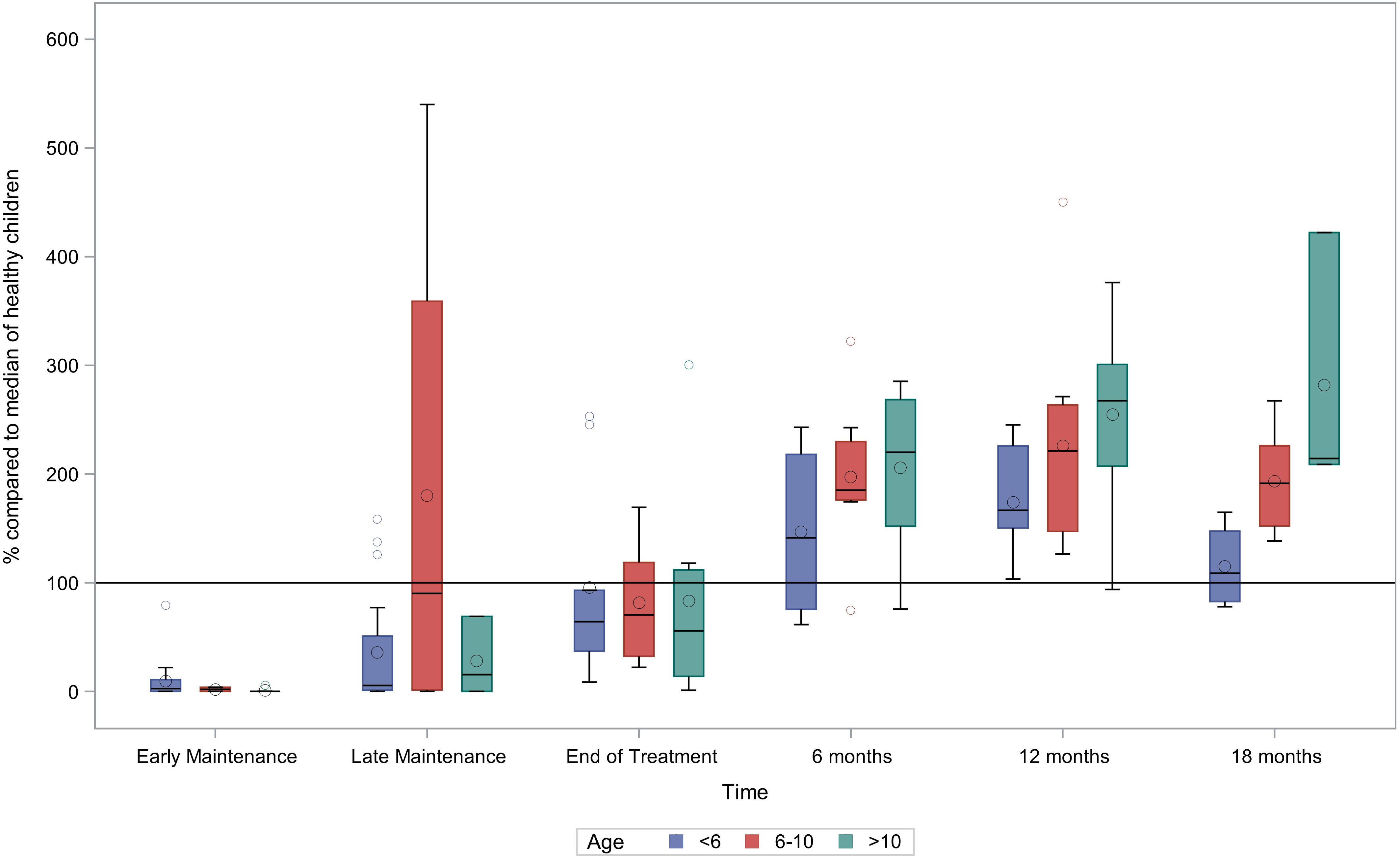

P-values by age group and time

	Early Maintenance	Late Maintenance	End of Treatment	6 months	12 months	18 months
<6	<0.001**	<0.001**	0.570**	0.026*	0.026*	0.496*
6-10	0.500**	0.999**	0.365**	0.003*	0.003*	0.001*
>10	0.063**	0.250**	0.329**	0.005*	0.005*	0.101*

*Wilcoxon signed-rank test of the naïve B cells and median naïve B cells of healthy children, at two-sided significance level of 5%

**Paired t-test of the naïve B cells and median naïve B cells of healthy children, at two-sided significance level of 5%

Note: please refer to Supplementary Table 1 for sample size information
