# Supplementary material for: Immune reconstitution in children following chemotherapy for acute leukemia
Source: EJHaem. 2020 Jun 10;1(1):142–51. doi: 10.1002/jha2.27 (PMC9176016; doi:10.1002/jha2.27)
Supplement: Supplementary file 9 — SUPPORTING INFORMATION [file JHA2-1-142-s006.docx]

**Supplementary Table 1 -** Number of sample results used in supplementary figures

| Figure | Measure | Early maintenance | Late maintenance | End of treatment | 6 months | 12 months | 18 months |
| --- | --- | --- | --- | --- | --- | --- | --- |
| S1a | lgG % within normal range | 38 | 37 | 39 | 35 | 30 | 29 |
| S1a | lgA % within normal range | 38 | 37 | 39 | 35 | 28 | 29 |
| S1a | lgM % within normal range | 38 | 37 | 39 | 35 | 28 | 29 |
| S1b | lgG median | 38 | 37 | 39 | 35 | 30 | 29 |
| S1b | lgA median | 38 | 37 | 39 | 35 | 28 | 29 |
| S1b | lgM median | 38 | 37 | 39 | 35 | 28 | 29 |
| S2a | lgG1 % within normal range | 35 | 37 | 37 | 30 | 30 | 32 |
| S2a | lgG2 % within normal range | 35 | 37 | 38 | 33 | 29 | 32 |
| S2a | lgG3 % within normal range | 35 | 37 | 34 | 29 | 28 | 31 |
| S2a | lgG4 % within normal range | 35 | 37 | 36 | 31 | 29 | 32 |
| S2b | lgG1 median | 35 | 37 | 37 | 30 | 30 | 32 |
| S2b | lgG2 median | 35 | 37 | 38 | 33 | 29 | 32 |
| S2b | lgG3 median | 35 | 37 | 34 | 29 | 28 | 31 |
| S2b | lgG4 median | 35 | 37 | 36 | 31 | 29 | 32 |
