# Supplementary material for: Immune reconstitution in children following chemotherapy for acute leukemia
Source: EJHaem. 2020 Jun 10;1(1):142–51. doi: 10.1002/jha2.27 (PMC9176016; doi:10.1002/jha2.27)
Supplement: Supplementary file 8 — SUPPORTING INFORMATION [file JHA2-1-142-s007.pdf]

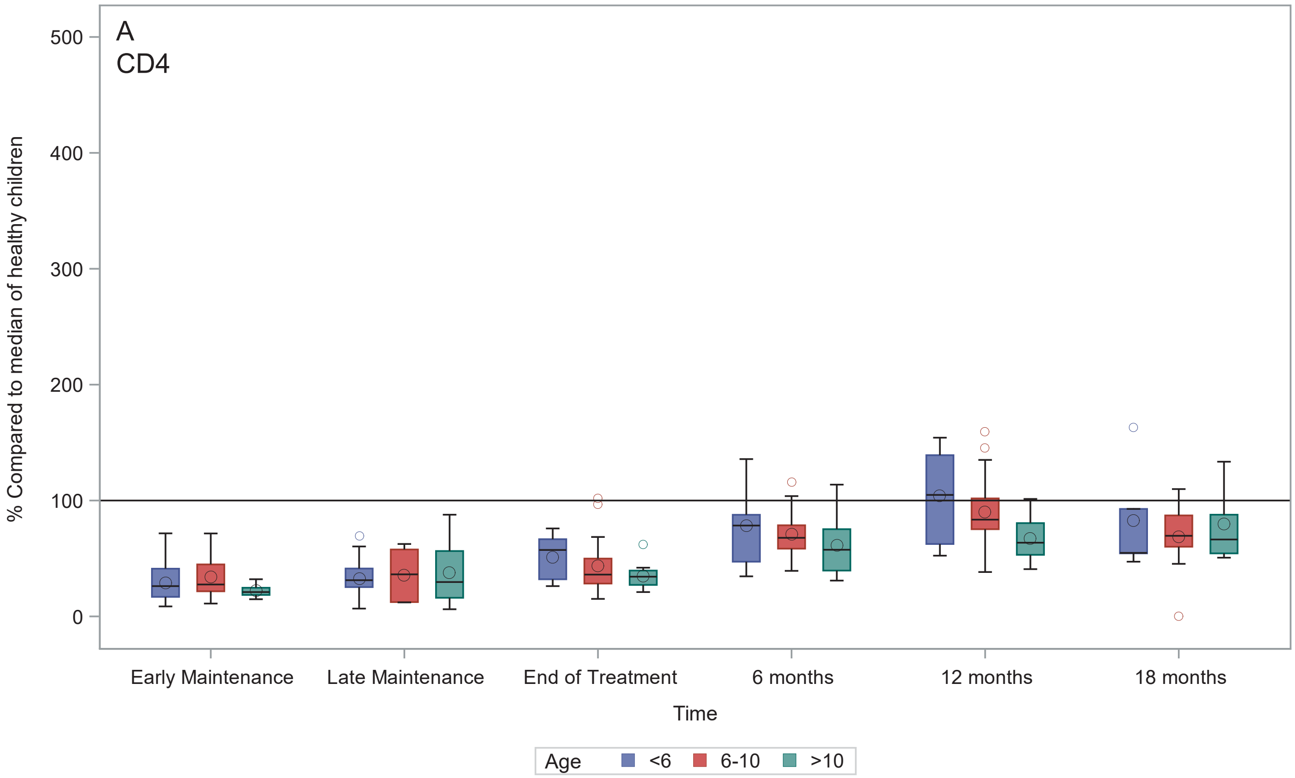

P-values by age group and time

	Early Maintenance	Late Maintenance	End of Treatment	6 months	12 months	18 months
<6	<0.001*	<0.001*	<0.001*	0.128*	0.734**	0.410*
6-10	<0.001*	<0.001*	<0.001*	<0.001*	0.305**	<0.001*
>10	<0.001*	0.003*	<0.001*	<0.001*	0.016**	0.087*

*Wilcoxon signed-rank test of the CD4 T cells and median CD4 T cells of healthy children, at two-sided significance level of 5%
**Paired t-test of the CD4 T cells and median CD4 T cells of healthy children, at two-sided significance level of 5%
Note: please refer to Supplementary Table 1 for sample size information

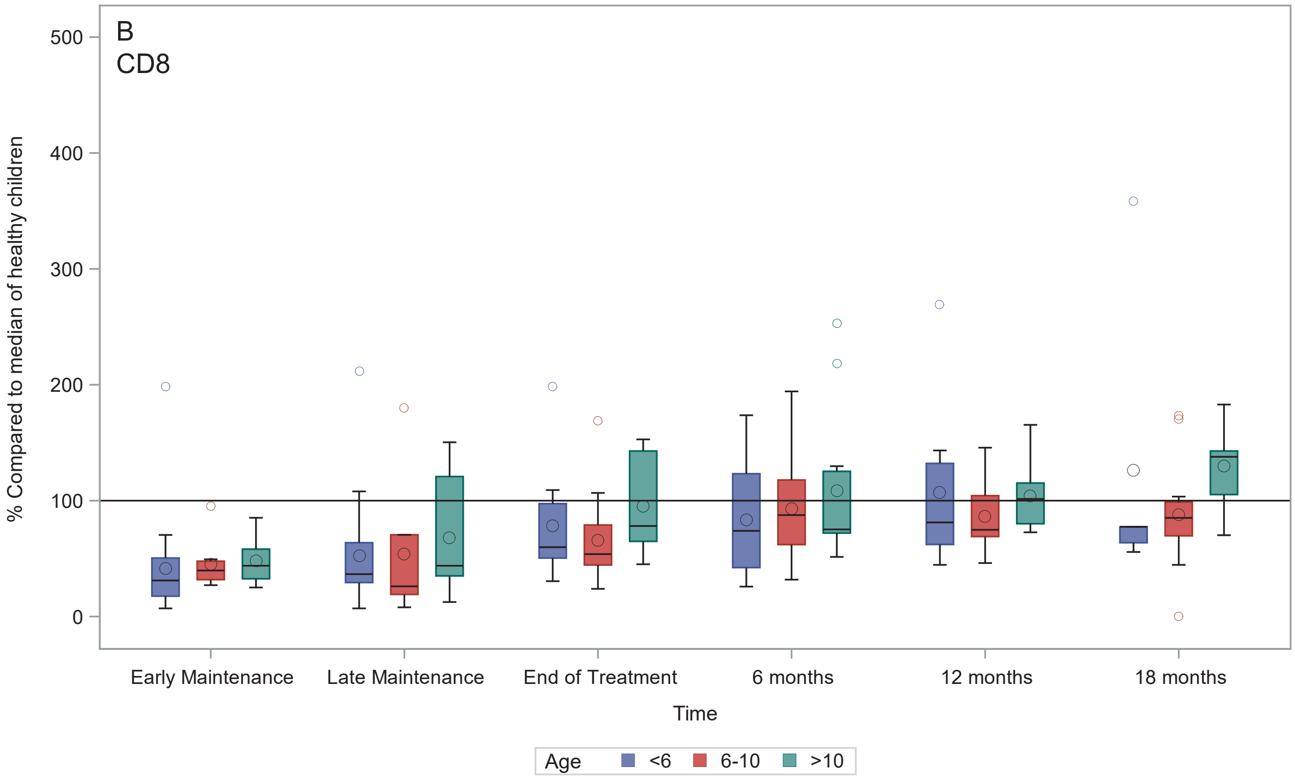

P-values by age group and time

	Early Maintenance	Late Maintenance	End of Treatment	6 months	12 months	18 months
<6	<0.001**	<0.001**	<0.114*	0.375**	0.758**	0.563**
6-10	0.008**	0.156**	0.001*	0.325**	0.123**	0.081**
>10	0.031**	0.219**	0.569*	0.722**	0.945**	0.020**

*Wilcoxon signed-rank test of the CD8 T cells and median CD8 T cells of healthy children, at two-sided significance level of 5%
**Paired t-test of the CD8 T cells and median CD8 T cells of healthy children, at two-sided significance level of 5%
Note: please refer to Supplementary Table 1 for sample size information
